# Statistical aspects of the TNK-S2B trial of tenecteplase versus alteplase in acute ischemic stroke: an efficient, dose-adaptive, seamless phase II/III design

**DOI:** 10.1177/1740774511410582

**Published:** 2011-08

**Authors:** Bruce Levin, John LP Thompson, Bibhas Chakraborty, Gilberto Levy, Robert MacArthur, E Clarke Haley

**Affiliations:** a Department of Biostatistics, Mailman School of Public Health, Columbia University, New York, NY, USA; b Research Pharmacy, Columbia University, New York, NY, USA; c Department of Neurology, University of Virginia, Charlottesville, VA, USA

## Abstract

***Background*** TNK-S2B, an innovative, randomized, seamless phase II/III trial of tenecteplase *versus* rt-PA for acute ischemic stroke, terminated for slow enrollment before regulatory approval of use of phase II patients in phase III.

***Purpose*** (1) To review the trial design and comprehensive type I error rate simulations and (2) to discuss issues raised during regulatory review, to facilitate future approval of similar designs.

***Methods*** In phase II, an early (24-h) outcome and adaptive sequential procedure selected one of three tenecteplase doses for phase III comparison with rt-PA. Decision rules comparing this dose to rt-PA would cause stopping for futility at phase II end, or continuation to phase III. Phase III incorporated two co-primary hypotheses, allowing for a treatment effect at either end of the trichotomized Rankin scale. Assuming no early termination, four interim analyses and one final analysis of 1908 patients provided an experiment-wise type I error rate of <0.05.

***Results*** Over 1,000 distribution scenarios, each involving 40,000 replications, the maximum type I error in phase III was 0.038. Inflation from the dose selection was more than offset by the one-half continuity correction in the test statistics. Inflation from repeated interim analyses was more than offset by the reduction from the clinical stopping rules for futility at the first interim analysis.

***Limitations*** Design complexity and evolving regulatory requirements lengthened the review process.

***Conclusions*** (1) The design was innovative and efficient. Per protocol, type I error was well controlled for the co-primary phase III hypothesis tests, and experiment-wise. (2a) Time must be allowed for communications with regulatory reviewers from first design stages. (2b) Adequate type I error control must be demonstrated. (2c) Greater clarity is needed on (i) whether this includes demonstration of type I error control if the protocol is violated and (ii) whether simulations of type I error control are acceptable. (2d) Regulatory agency concerns that protocols for futility stopping may not be followed may be allayed by submitting interim analysis results to them as these analyses occur.

## Introduction

The TNK-S2B trial [1] was an innovative, multi-center, double blind, randomized, seamless phase II/III study of intravenous tenecteplase (TNK) *versus* standard-dose intravenous alteplase (rt-PA at 0.9 mg/kg) for treatment of patients with acute ischemic stroke within 3 h of onset. A key motivating factor for development of tenecteplase was to produce a molecular variant of rt-PA that would reduce the risk of symptomatic intracranial hemorrhage (ICH) while retaining clinical efficacy. The phase II component employed an adaptive sequential dose selection procedure to choose a preferred dose of tenecteplase, using an early (24 h) assessment of *major neurological improvement* (MNI) balanced against occurrence of symptomatic ICH. Once a preferred tenecteplase dose was established, it was moved forward in the phase III component to compare with standard-dose rt-PA. Decision rules comparing the selected tenecteplase dose and rt-PA on safety and efficacy outcomes were devised to yield a clear recommendation to either stop the trial for futility at the end of phase II, or continue into phase III. The trial was prematurely terminated for slow enrollment after 112 patients had been randomized at 8 clinical centers between 2006 and 2008. At that point, the proposal to include patients enrolled in the phase II portion of the trial in the phase III analysis had not received regulatory approval because of concerns regarding control of the type I error rate.

Although the trial results were insufficient to establish promise or futility [1], its novel adaptive design, with substantial provision for the control of type I error, is nevertheless of interest, given current discussion of draft FDA guidelines on adaptive designs [2]. This article has two goals. The first is to review the key design features of the trial, and the design and results of its comprehensive type I error rate simulation studies. The second is to discuss issues raised during the regulatory review of the design, which focused on the type I error simulations, and their implications for gaining approval for use of this type of adaptive design in the future.

## The TNK-S2B Design

The TNK-S2B trial incorporated three main design features: (1) a *sequential selection procedure* for choosing one tenecteplase dose from among three candidate doses (0.10, 0.25, and 0.40 mg/kg), by the *sequential elimination* of inferior dose arms, potentially leading to a dose selection decision at an early stage of phase II; (2) at the end of phase II, a *preliminary comparison of the clinical efficacy and safety endpoints* from patients treated with the selected dose of tenecteplase to those from patients treated with rt-PA, to determine promise or futility for continuing into phase III; and (3) if the results were promising, continuation of the study, with additional clinical sites, to compare the selected dose of tenecteplase with rt-PA in phase III, treating the decisions at the end of phase II as the first interim analysis of phase III. We provide details about each of these features.

### Sequential selection procedure for the best dose of tenecteplase

The selection procedure was a truncated Levin-Robbins sequential elimination procedure [3,4] based on a rapid-response, ordered trichotomous outcome determined within 24 h of randomization. The best category was MNI, defined as a ≥8-point improvement from baseline on the NIH Stroke Scale [5] or a score of 0 at 24 h after stroke onset. The worst category was symptomatic ICH within 24 h of stroke onset. The intermediate category was *neither* MNI nor ICH (NEI). Patients were randomized in permuted blocks of size 4 matched by clinical site (quadruplets) to rt-PA or one of the three doses of tenecteplase. Although patients were also randomized concurrently to rt-PA to preserve the blinding and to eliminate other possible temporal biases, only the tenecteplase arms were involved in the selection procedure. Once a rapid response was obtained from each of the three patients assigned to the tenecteplase doses in a matched quadruplet, their data were entered into an ongoing *sequential cumulative sum scoring system*. This added the value 2 to the cumulative sum for any dose arm for a rapid-response outcome of MNI, the value 1 for a rapid-response outcome of NEI, and the value 0 for a rapid-response outcome of ICH. The first time the cumulative sum for any arm(s) with the *largest* sum was (were) *6 points ahead* of the cumulative sum for any arm(s) with the *smallest* sum, the arm(s) with the smallest sum was (were) to be *eliminated* from the study, meaning that those arms were no longer to be considered candidates for selection and patients were no longer to be randomized to those arms. If one dose arm was eliminated at this time, patients were to be randomized to the remaining arms (and to rt-PA), and the procedure was to proceed with their corresponding cumulative sums continued at their current tallies, until a second and final arm was eliminated. Once the second arm had been eliminated, the procedure was to terminate and the remaining arm was to be selected as the preferred dose of tenecteplase. If initially two arms were eliminated simultaneously, the procedure was to terminate then and select the remaining arm as the preferred dose of tenecteplase. If dose selection occurred after complete observation of the rapid responses from fewer than 100 tenecteplase-matched sets, randomization continued to either the selected dose of tenecteplase or rt-PA until a total of 100 patients on each treatment had been randomized, at which time the clinical assessment for promise or futility was to be conducted. If the dose selection occurred after complete observation of the rapid responses from between 100 and 150 tenecteplase-matched sets, randomization was to stop and the clinical assessment for promise or futility was to take place then. If no dose selection had yet occurred after complete observation of the rapid responses from 150 tenecteplase-matched sets, the procedure was to be truncated, i.e., randomization would stop, and the dose selection was to be completed in conjunction with the clinical assessment for promise or futility as described below. The selection criterion of a lead of 6 was chosen to achieve a *probability of correct selection* of ≥0.8 under the design alternative probabilities of 0.31, 0.21, and 0.21 for the rapid-response outcome of MNI for the three tenecteplase doses, assuming a common probability of ICH of 0.06 for each dose. A phase I pilot study of tenecteplase [6] had shown substantially larger differences in the proportion of subjects with MNI between tenecteplase doses: 36% of subjects given 0.1 mg/kg had MNI compared with 16% of subjects given 0.4 mg/kg. The literature also showed that ICH rates of approximately 6% are typical with rt-PA administration [7]. The truncation point was selected small enough to achieve a feasible maximum recruitment, yet sufficiently large to preserve the probability of correct selection.

The operating characteristics of the selection procedure were estimated by simulation based on 100,000 replications for each scheme, and are presented in Table 1. *P*[cs] refers to the probability of correct selection, i.e. reaching a final elimination by the stated criterion with a correct selection of the best dose at or before the 150th matched set. The value for *P*[cs] does not include any correct selections that might also occur at truncation time by clinical decision criteria should a final winner not be declared by 150 matched sets. *E*[min(*N*(1),*m*)] refers to the expected time of first elimination or 150 triplets, whichever occurs first. This gives the average number of matched triplets until the earlier of the first dose elimination or the end of the phase II trial; multiplying by 3 gives the number of patients randomized to this point in the procedure. *E*[min(*N*,*m*)] refers to the expected time of final elimination or 150 matched sets, whichever occurs first. This gives the average number of matched sets until a dose is selected or 150 matched sets have been randomized, whichever occurs first. One cannot multiply this number by 3 to get the total number of patients in the selection phase because of the possibility of early elimination of a dose. We therefore provide another operating characteristic, *E*[*T*], the expected total number of patients randomized in the selection phase. Also, provided are the median and modal numbers of matched sets (approximate to the nearest integer). Finally, *P*[no winner] refers to the probability that truncation time will arrive without achievement of the stated criterion for a final dose selection. In symbols, *P*[no winner] = *P*[*N* > 150]. In all cases, the probability of selecting an incorrect dose is given by 1 − *P*[cs] − *P*[no winner]. Table 1 shows the operating characteristics of different schemes in which one dose is superior and the two inferior doses are equal in probabilities of MNI and symptomatic ICH; this is often called a ‘least favorable configuration.’ The ‘best’ dose in Table 1 appears in the first row of each scheme. See Appendix (supplementary material) for the operating characteristics under five other schemes of interest.

**Table 1:** Operating characteristics of the selection procedure based on 100,000 simulations for each scheme under the least favorable configuration

	Scheme (%MNI – %ICH for three doses)				
	36% – 6%	36% – 6%	31% – 6%	31% – 6%	26% – 6%
	16% – 6%	16% – 2%	21% – 6%	21% – 2%	26% – 6%
	16% – 6%	16% – 2%	21% – 6%	21% – 2%	26% – 6%
*P*[cs]^a^	0.976	0.958	0.802	0.646	0.297
*E*[min(*N*(1),*m*)]	22.5	27.6	30.7	38.5	35.4
*E*[min(*N*,*m*)]	35.9	43.7	59.3	73.6	74.2
Median[*N*]	31	37	50	65	65
Mode[*N*]	21	24	29	40	35
*E*[*T*]	94.4	115.0	149.3	185.7	183.7
*P*[no winner]	0.0026	0.0093	0.041	0.107	0.110

a When the doses have equal probability of MNI and symptomatic ICH (as in the scheme presented in the last column), selection of any of the three doses is ‘correct’ with respect to the probability of MNI net of symptomatic ICH. In this case, the first row gives the probability of selecting the first listed doses. Exactly the same figure applies to the other two doses.

### Preliminary comparisons of the clinical efficacy and safety endpoints

The preliminary assessment for promise or futility at the end of phase II was based on the modified Rankin scale [8] observed 3 months after randomization, trichotomized into three ordered categories. The best category was Rankin score 0 or 1 (*good* outcome); the worst category was Rankin score 4, 5, or 6 (*poor* outcome, including death); and the intermediate category was Rankin score 2 or 3 (*neither*, i.e., neither *poor* nor *good* outcome). There were three sets of clinical decision rules for declaring promise or futility, depending on the relative safety profile of (the selected dose of) tenecteplase compared to rt-PA.

*Scenario 1: Tenecteplase showed a lower symptomatic ICH rate than rt-PA, defined as tenecteplase having at least 2 fewer symptomatic ICHs than rt-PA on the rapid-response outcome*. In this situation, declare tenecteplase promising if the observed proportion of patients with poor outcome is less than or equal to that of rt-PA*.* If the proportion with poor outcome on tenecteplase is greater than on rt-PA, declare further study unpromising. In addition, and consistent with the interim monitoring plan for safety and efficacy, declare further study unpromising if the proportion of good outcomes for tenecteplase is significantly less than for rt-PA at the nominal two-tailed 0.001 level.

*Scenario 2: The rate of symptomatic ICH within 24 h for tenecteplase was effectively the same as for rt-PA*, *defined as tenecteplase having the same number of ICHs as rt-PA or differing at most by plus or minus one ICH.* In this situation, declare tenecteplase promising if the proportion of patients on tenecteplase with poor Rankin outcome is at least 8 percentage points lower than on rt-PA (i.e., an arithmetic difference of 0.08). If not, declare further study unpromising. As in Scenario 1, also declare further study unpromising if the proportion of good outcomes for tenecteplase is significantly less than for rt-PA at the nominal two-tailed 0.001 level.

*Scenario 3: Tenecteplase showed a higher symptomatic ICH rate than rt-PA, defined as tenecteplase having two or more ICHs than rt-PA*. In this situation, declare further study unpromising.

These rules for declaring promise or futility were clinical decision rules and were not based on statistical significance criteria (except where the interim monitoring plan would suggest that the DSMB consider early stopping of the study). While somewhat arbitrary, they reflected the clear wishes of the clinical investigator, and provided unambiguous grounds for the required clinical decision making at the end of phase II. They can also be viewed as another statistical selection procedure: at this point in the trial, we would need to select between two alternative courses – ‘to go’ or ‘not to go’ on to phase III – with a procedure that would provide a high probability of correct selection in the event there was a truly superior choice.

It is possible that the selection procedure might observe 150 matched sets without arriving at a selection decision. In that case, the following clinical criteria would be used to select a dose from the remaining two or three competing arms and determine promise or futility.

*Criterion 1*: Select the dose that allows continuation into phase III based on the futility criteria specified above. If no dose would allow continuation of the study based on the futility criteria, further study with any of the three doses would be declared unpromising.

*Criterion 2*: If under Criterion 1 more than one dose would allow continuation of the trial, select the tenecteplase dose with the *lowest ICH rate*.

*Criterion 3*: If under Criterion 2 more than one dose would allow continuation of the trial, select the dose with the *lowest proportion of poor outcomes*.

*Criterion 4*: If under Criterion 3 more than one dose would allow continuation of the trial, select the dose with the *highest proportion of good outcomes*.

See Appendix for further discussion.

### Continuation of the study in phase III

If the selected dose of tenecteplase showed promise, a total of at most 1908 patients were to be randomized in phase III to the selected dose of tenecteplase or rt-PA (954 per group, including the patients studied in these two arms during phase II). The primary endpoint for phase III was the trichotomized Rankin score. Two co-primary null hypotheses were to be tested in phase III: (1) The proportion of *poor* outcomes with tenecteplase at the selected dose did not differ from the proportion of poor outcomes with rt-PA. (2) The proportion of *good* outcomes with tenecteplase at the selected dose did not differ from the proportion of good outcomes with rt-PA. Each hypothesis was to be tested using the Mantel–Haenszel score test, stratified by site, with 1/2-continuity correction. The two hypotheses were to be tested at the overall two-tailed *α* = 0.05 level using the Holm step-down procedure [9], which controls the probability of making one or two type I errors at no more than 0.05. The planned sample size of 1,908 (954 per group) would provide 90% power to detect a ≥8% reduction in poor outcome without a reduction in good outcome, or 89% power to detect an 8% increase in good outcome without an increase in poor outcome.

We formulated the two co-primary hypotheses to allow for a treatment effect at either end of the Rankin scale. This also facilitated identifying the ‘win/lose’ situations for tenecteplase, as required by regulatory reviewers. For example, if tenecteplase were no better than a placebo, it would reduce the proportion of poor outcomes due to ICH, although it would also have poor efficacy compared to rt-PA. This would not be a ‘win’ situation.

The phase III trial was to incorporate four formal interim analyses and one final analysis. The first interim analysis was to take place at the end of phase II after enrollment of between 200 and 300 patients in the two phase III arms (between 100 and 150 patients per arm, and not counting the other arms used in the selection stage). The second, third, and fourth interim analyses were to take place after follow-up was completed for a total of 500, 1,000, and 1,500 patients, respectively. The terminal analysis was to take place after a total of 1908 patient observations were complete, assuming no early termination.

**Figure 1:**
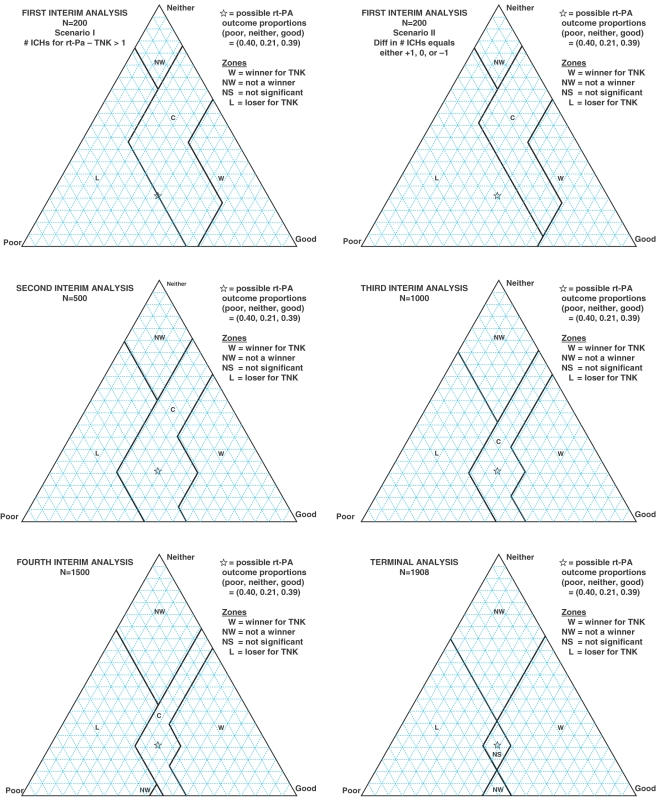
Graphical representation of the win-lose-type situations for tenecteplase in the interim analyses as well as the terminal analysis, using barycentric coordinates

Figure 1 contains a graphical representation of the win-lose-type situations for tenecteplase in the interim analyses and the terminal analysis, using barycentric coordinates. Any point in the triangle represents a triplet consisting of the probabilities of poor, neither, and good outcome; the perpendicular distance of the point from any one of the three sides represents the probability of the outcome denoted by the vertex opposite that side.

At each interim analysis, formal tests of the two primary hypotheses were to be conducted, each at the two-tailed *α* = 0.001 level. Assuming both null hypotheses to be true, rejection of either at any interim analysis would constitute at least one type I error. At the terminal analysis, assuming no earlier stopping, each primary hypothesis was to be tested at the nominal *α* = 0.025 level with 1/2-continuity correction.

Type I error control in seamless phase II/III trials poses unresolved issues, particularly when, as here, phase II involves a selection procedure rather than a hypothesis test. Two are particularly important in the current case.

#### Multiple testing

No correction for multiple testing is needed because the phase III component of the trial does not compare each competing dose of tenecteplase with rt-PA; it performs *only one* hypothesis test of the selected dose of tenecteplase *versus* rt-PA (for each of the two co-primary endpoints). The inferior tenecteplase doses are eliminated in phase II by a selection procedure, not a hypothesis test against rt-PA; moreover, the selection procedure uses an outcome different from (although correlated with) that in phase III. Thus, although the hypothesis tested in phase III is determined by the selection procedure, there is only one hypothesis test for each co-primary endpoint. Since no more than a single type I error can be committed, the need for adjustment for multiple testing does not arise.

#### Selection bias

The selection procedure does slightly inflate the type I error rate. However, we show in a fixed sample size simulation study (see below, and the Appendix) that without stopping for futility or efficacy in interim monitoring, this inflation is more than compensated for by the 1/2-continuity correction’s reduction in the type I error rate. We also show below, in a group sequential study, that the clinical criteria for futility stopping even with interim monitoring reduce the type I error rate further below nominal levels. Given this, no correction for selection bias resulting from the selection procedure is necessary.

## Design of the type I error rate simulation studies

Given the complex design of TNK-S2B, simulation studies were required to demonstrate that the statistical analysis plan specified above does in fact control the type I error rate for the phase III trial at or below nominal levels. We conducted two such studies. One, the Group Sequential (GS) study, is presented here. A second, the Fixed Sample Size (FSS) study, is included as an Appendix. As argued above, there was only one hypothesis test contemplated from the beginning of phase II to the end of phase III, that of the selected tenecteplase dose *versus* rt-PA, and hence the type I error rate we refer to here is indeed an unconditional, experiment-wise error rate and not conditioned on the identity of the dose selected.

The FSS study considers a simplified version of the TNK-S2B trial design in which dose selection is possible; there is no examination of clinical outcome data for promise or futility; and the trial proceeds to a single final analysis with a total of 1,908 patients, 954 in the selected tenecteplase arm and 954 in the rt-PA arm, with no stopping for futility or for strong showing of interim efficacy. Its purpose is to highlight the simultaneous effects of the dose selection from stage 1 and the 1/2-continuity correction, which would otherwise be dominated by the larger effect of the examination for promise or futility and the effect of interim monitoring. See Appendix for details.

### The GS simulation study

The purpose of the GS study is to simulate the full TNK-S2B trial, including the dose selection, examination for promise or futility, and phase III interim monitoring features, to estimate their combined impact on the type I error rates. All replications of the GS study are used in the estimation of the error rates. Note that according to the design, if the actual trial stopped at the end of phase II for futility because a selected dose lacked promise, or if there was a failure to select a tenecteplase dose because all competing doses lacked promise at truncation time, there would be no phase III trial and hence no declaration of significance and no type I errors under the null hypothesis (unless a dose had a significantly different probability of poor or good outcome compared to rt-PA at the 0.001 level). Thus, in the GS study, if a particular simulation under a given null hypothesis scheme stops for futility because a selected dose lacks promise (without attaining statistical significance at the two-tailed 0.001 level for either endpoint), we count the simulation as contributing no type I error. If the simulation results in a failure to select a tenecteplase dose because all competing doses lack promise at truncation time, we choose one dose at random to test for statistical significance at the two-tailed 0.001 level for both poor and good outcomes, after which the simulated trial stops. On the other hand, if at any interim analysis the two-tailed 0.001 significance level is attained for either poor or good clinical outcome, or at the terminal analysis the two-tailed 0.025 significance level is attained for either poor or good clinical outcome, we count the simulation as contributing at least one type I error.

Each simulation study used 40,000 replications under each of 1000 different null hypothesis schemes described below. Note that the Mantel–Haenszel score test would have type I error rates almost identical to that of a simple comparison of pooled proportions, because in this trial the site-stratified randomization procedure rendered the stratification factor orthogonal to the treatment assignment under the null hypothesis. Therefore, for feasibility of the present type I error rate studies, we used only the pooled *z*-score test for the equality of two proportions with 1/2-continuity correction for each primary hypothesis.

### Specification of distribution schemes

**Figure 2:**
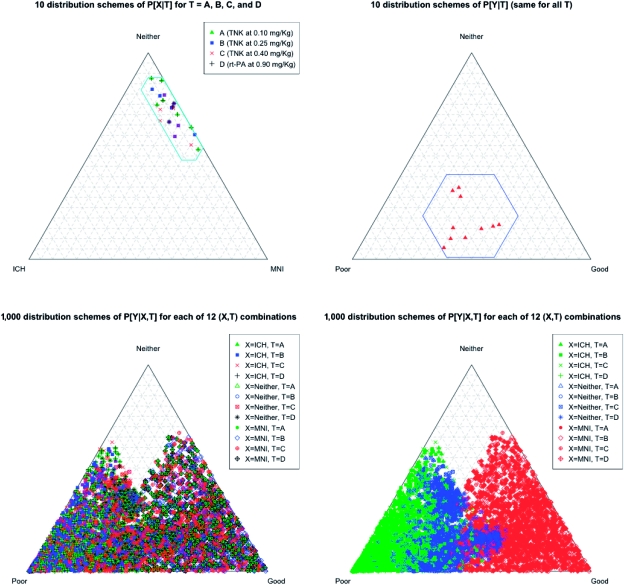
Graphical representation of the different distribution schemes in terms of barycentric coordinates: (a) 10 marginal conditional distributions of *X*|*T*, for *T* = A, B, C, D, along with the region of clinical interest; (b) 10 marginal conditional distributions of *Y*|*T* (same for all *T* under the null hypothesis), along with the region of clinical interest; (c) 1,000 distributions of *Y*|*X*, *T* for each of 12 (*X*, *T*) combinations, where different colors represent different values of *T*; (d) 1,000 distributions of *Y*|*X*, *T* for each of 12 (*X*, *T*) combinations, where different colors represent different values of *X*, and which is consistent with the clinical monotonicity constraint

The simulation of both rapid-response outcomes and 3-month Rankin outcomes requires specification of the joint distribution of two trichotomous random variables, i.e., a 3 × 3 table of joint probabilities, for each treatment arm. We call such a set of four 3 × 3 tables a *distribution scheme*; 1000 different distribution schemes were used in the simulation studies. Generation of these distribution schemes is described in detail in the Appendix. In the simulation studies, our goal was to produce a set of distributions that would cover a portion of the parameter space that was of direct clinical interest to the TNK-S2B trial, and that the portion covered was sufficiently broad as to represent type I error rates accurately over the entire theoretical parameter space. Let the three-category rapid response be denoted by *X*, taking values of 0 for ICH, 1 for neither MNI nor ICH, and 2 for MNI. Let the trichotomized 3-month Rankin scale for the clinical outcome be denoted by *Y*, taking values of 0 for poor outcome, 1 for neither poor nor good outcome, and 2 for good outcome. Let the doses of tenecteplase be labeled A, B, and C, corresponding to 0.1, 0.25, and 0.40 mg/kg, respectively, and let dose D refer to rt-PA. Let *T* denote any of these four treatment arms. It is most convenient to determine the joint distribution of (*X*, *Y*) by first specifying *P*[*X*|*T*], and then specifying *P*[*Y*|*X*, *T*]. Once these three-component vectors of probabilities are determined, random realizations of the pair (*X*, *Y*) can be produced by generating a trinomial response *X* following *P*[*X*|*T*], and then by generating another trinomial response *Y* following *P*[*Y*|*X*, *T*]. In the simulation studies, 10 different marginal conditional distributions for *X* given T were selected at random in a manner described in the Appendix; see Figure 2(a) for visualizing these distributions. For each one of these, 10 different marginal distributions for *Y* given *T* (identical for each *T* under the null hypotheses) were generated randomly (Figure 2(b)); and for each of the 10 × 10 = 100 pairs of marginal distributions for *X* and *Y*, 10 different conditional distributions for *Y* given *X* and *T* were generated randomly, subject to the *marginalization constraint* that the weighted average of *P*[*Y*|*X*, *T*] using weights *P*[*X*|*T*] agree with the given distribution *P*[*Y*|*T*], together with a *clinical monotonicity constraint* that *P*[*Y* = poor|*X*, *T*] is the greatest when *X* = ICH and least when *X* = MNI, and similarly, *P*[*Y* = good|*X*, *T*] is the least when *X* = ICH and the greatest when *X* = MNI. Thus, 10 × 10 × 10 = 1000 different distribution schemes were employed for the simulations. Figure 2(c) contains a graphical representation of the complete list of distributions, using barycentric coordinates, displaying a uniform distribution of *T* over the lower portion of the triangle. Figure 2(d) shows a visual effect of the clinical monotonicity constraint. As stated above, we simulated 40,000 trial replications for each distribution scheme. Each replication generated random samples of up to 150 pairs of outcomes (*X*, *Y*) for each member of a quadruplet or matched set for the selection stage, and additional (*X*, *Y*) pairs to make up the total sample size of 1908 clinical outcomes for the phase III trial.

## Results

In the GS study, the type I errors were all well below their nominal levels, by about one-third. Table 2 shows that the average two-tailed type I error rate (averaged across all 1,000 distribution schemes) was approximately 0.009 for both poor and good outcomes. Among the 2,000 hypothesis tests conducted among the 1,000 distribution schemes, the maximum estimated type I error was approximately 0.019. The third panel of Table 2 shows that the average of the overall type I error rate for the two primary outcomes (i.e., the probability of at least one type I error) was approximately 0.018, with maximum value approximately 0.038. The type I error rate is evidently under good control.

**Table 2:** Descriptive statistics of the distribution of type I errors in the GS study across 1,000 schemes

Mean	Median	Maximum	Minimum	Lower quartile	Upper quartile	Range	Std Dev.
Analysis variable: type I error for poor outcome							
0.0091	0.0097	0.0194	0.0012	0.0053	0.0123	0.0182	0.0045
Analysis variable: type I error for good outcome							
0.0088	0.0096	0.0192	0.0010	0.0046	0.0120	0.0182	0.0046
Analysis variable: type I error for either poor or good outcome (overall error)							
0.0179	0.0194	0.0380	0.0024	0.0098	0.0243	0.0356	0.0091

These results show that the inflation in the type I error rates caused by the dose selection and repeated looks at the data in interim analyses is more than offset by the reduction in the type I error rate caused by implementing the clinical stopping rules for futility at the first interim analysis. In the GS study, selection in the phase II component somewhat elevates the probability of declaring the selected dose of tenecteplase significantly better than rt-PA with respect to poor outcome, while it simultaneously decreases somewhat the probability of declaring the selected dose of tenecteplase significantly worse than rt-PA. The average type I error rate in the former tail was 0.0069 while that in the latter tail was 0.0022. Analogous results were obtained for good outcome. The average type I error rate for declaring the selected dose of tenecteplase significantly better than rt-PA with respect to good outcome was 0.0061 while the average type I error rate for declaring the selected dose of tenecteplase significantly worse than rt-PA was 0.0027. Note how the asymmetrical allocations of type I error counterbalance each other, such that the sum of the error rates in the two tails are within nominal levels. It is entirely acceptable for a two-tailed test to employ an asymmetrical allocation of total type I error in the two tails, so long as the total type I error rate is within nominal levels [10]. Similar results were obtained in the FSS study, and are described in detail in the Appendix.

## Discussion

The TNK-S2B trial employed an innovative, randomized, seamless phase IIB/III design to test tenecteplase *versus* standard-dose rt-PA in the treatment of patients with acute ischemic stroke within 3 h of onset. For the phase II part of the trial, we chose an adaptive sequential dose selection procedure that employed a rapid assessment of MNI at 24 h balanced against occurrence of symptomatic ICH to choose among three different doses of tenecteplase.

Regulatory reviewers questioned three aspects of the control of the type I error rate. The first concerned whether or not multiple comparisons techniques were required given that the trial began with three doses of tenecteplase, and whether or not an adjustment for selection effects was needed. We have addressed those concerns above.

Reviewers were further concerned that the type I error rate would not be controlled under schemes which would not occur under the protocol, e.g., if the trial were continued into phase III absent clinical criteria for promising efficacy. We understand the concern, in that noncompliance with prespecified protocols is a major problem when it occurs, as it often has. However, this would have been in violation of the clearly specified trial protocol, which stated the futility stopping requirements as *rules* rather than *guidelines*. It has been accepted in the ongoing dialog between researchers and regulators that adaptive designs must be prespecified rather than *ad hoc*. This presupposes and requires respect for the prespecified protocol, under which the type I error rate is computed. Ultimately, one cannot assure type I error control under arbitrary protocol violations. This applies with equal force to traditional as well as adaptive trial designs.

Finally, a reviewer was doubtful whether any amount of simulations could demonstrate adequate control of the type I error rate. However, simulations are recognized under the FDA draft guidance document [2], and given the coverage of the parameter space in ours, it seemed to us unreasonable to sustain such doubt. Clarification is needed whether control of the error rate must be demonstrated by theorems or can be demonstrated by simulation. In addition, a sensitive reviewer of this article suggested that the regulatory agency’s concern might be allayed by a commitment from the trial investigators to submit interim analysis results to it as these analyses occur. This proposal merits consideration.

In summary, the TNK-S2B trial design, while complex, was innovative and efficient. Its statistical analysis plan had great integrity. Under the protocol, it would have controlled the unconditional type I error rate below the nominal 0.05 level for the two primary hypothesis tests in phase III, and experiment-wise. The trial provides several lessons for adaptive designs. (a) Time must be allowed for iterative communications with regulatory reviewers from the first stages of the design and planning process, as the recent FDA draft guidance document stresses [2]. (b) Type I error control must be clearly demonstrated. (c) Greater clarity is needed on whether this includes demonstration that type I error control will be maintained if the protocol is violated, and on whether or not simulations are an acceptable form of demonstration. (d) Regulatory agency concerns that the protocol for futility stopping may not be followed may be allayed by a commitment to submit all interim analysis results to the regulatory agency as these analyses occur. Future trials with similar potential to TNK-S2B will have a greater probability of success if these issues can be successfully addressed.
